# A Thermo-Electro-Viscoelastic Model for Dielectric Elastomers

**DOI:** 10.3390/ma16175917

**Published:** 2023-08-29

**Authors:** Bao Qin, Zheng Zhong, Tong-Yi Zhang

**Affiliations:** 1 Research Institute of Interdisciplinary Science & School of Materials Science and Engineering, Dongguan University of Technology, Dongguan 523808, China; qinbao1194723766@163.com; 2 School of Science, Harbin Institute of Technology, Shenzhen 518055, China; 3 Advanced Materials Thrust and Sustainable Energy and Environment Thrust, The Hong Kong University of Science and Technology (Guangzhou), Guangzhou 511400, China; zhangty@shu.edu.cn

**Keywords:** dielectric elastomers, thermo-electro-viscoelasticity, finite deformation, thermodynamic consistency

## Abstract

Dielectric elastomers (DEs) are a class of electro-active polymers (EAPs) that can deform under electric stimuli and have great application potential in bionic robots, biomedical devices, energy harvesters, and many other areas due to their outstanding deformation abilities. It has been found that stretching rate, temperature, and electric field have significant effects on the stress-strain relations of DEs, which may result in the failure of DEs in their applications. Thus, this paper aims to develop a thermo-electro-viscoelastic model for DEs at finite deformation and simulate the highly nonlinear stress-strain relations of DEs under various thermo-electro-mechanical loading conditions. To do so, a thermodynamically consistent continuum theoretical framework is developed for thermo-electro-mechanically coupling problems, and then specific constitutive equations are given to describe the thermo-electro-viscoelastic behaviors of DEs. Furthermore, the present model is fitted with the experimental data of VHB4905 to determine a temperature-dependent function of the equilibrium modulus. A comparison of the nonlinear loading-unloading curves between the model prediction and the experimental data of VHB4905 at various thermo-electro-mechanical loading conditions verifies the present model and shows its ability to simulate the thermo-electro-viscoelastic behaviors of DEs. Simultaneously, the results reveal the softening phenomena and the instant pre-stretch induced by temperature and the electric field, respectively. This work is conducive to analyzing the failure of DEs in functionalities and structures from theoretical aspects at various thermo-electro-mechanical conditions.

## 1. Introduction

Electro-active polymers (EAPs) that deform under electric stimuli are emerging as promising materials for wide applications such as actuators and sensors [1]. Among various types of EAPs, dielectric elastomers (DEs) have attracted more attention in recent years due to their outstanding deformation abilities (about 50–380%), high energy density, fast response speed, light weight, and mechanical compliance [2], and have found their potential applications in bionic robots [3,4], biomedical devices [5,6], energy harvesters [7,8], flexible electronic devices [9,10] and so on. However, DEs may fail in their functionalities and structures when subjected to complicated or extreme environments. For example, a common design of DE film [11,12] or DE tube [13] may wrinkle or buckle because of the deformation induced by the interaction of charges with opposite signs on neighboring surfaces when an electric field is applied along the thickness direction. Besides, temperature fluctuations can influence the rate-dependent stress-strain relation and cause the electric and mechanical instability of DEs by changing their material properties [14,15]. Therefore, studying the thermo-electro-mechanically coupled process of DEs is of great significance in the development of DE materials and devices.

In the past few years, it has been long observed from experiments that the mechanical responses of DEs are highly rate-dependent [16,17]. The viscous dissipation energy, represented by the area between the loading and unloading curves, varies with the stretching rate. Later, a number of experimental tests, including single-step and multi-step relaxation tests and cyclic loading-unloading tests, were performed by Hossain and co-workers [18,19,20] to find that the viscoelastic behavior of the commonly used DE polymer VHB4910 was also sensitive to temperature and electric field. In particular, an increasing temperature can significantly soften the materials. Recently, Mehnert et al. [21] conducted more comprehensive tests, showing that temperature has a more pronounced effect on the viscoelastic behavior of DE polymer VHB4905 than an electric field.

At the same time, some phenomenological or physics-based models have also been established to simulate the viscoelasticity of DEs [22,23,24,25]. However, to the best of our knowledge, most of these models can only consider the thermo-mechanical or electro-mechanical behaviors of DEs. For example, Mehnert et al. [26] proposed an electro-viscoelastic model for DE polymer VHB4905 at room temperature without consideration of temperature fluctuation, while Alkhoury et al. [27] established a thermo-mechanical model to describe the significant viscoelasticity reduction of VHB4910 due to temperature increase, ignoring the effect of an electric field. Very few constitutive models were developed to describe the thermo-electro-viscoelastic behaviors of DEs. Although Mehnert et al. [28] proposed a theoretical model for dielectric elastomers with coupling thermo-electro-mechanical behaviors, for which temperature-dependent scaling functions were introduced to modify the elastic and viscous energy contributions in order to reflect the softening of materials induced by increasing temperature, the scaling functions were inconsistent and lacked a physical basis. Moreover, the used Yeoh-type and Neo-Hookean-type energy functions could not capture the strain-stiffening effect well, and the instant pre-stretch was not simulated in the theoretical model.

Therefore, in this paper, we aim to develop a thermo-electro-viscoelastic model for DEs at finite deformation and verify this model by using available experimental data from the literature. First, a thermodynamically consistent model for thermo-electro-mechanically coupling problems at finite deformation is established by considering the Gauss law and the electric contribution to the energy balance on the foundation of the classical thermo-mechanically coupling theoretical framework. Second, the viscoelasticity of DEs is described by a rheological model consisting of an elastic ground network and several parallel viscous subnetworks, whose elastic deformations are assumed to be incompressible and moduli to be temperature-dependent. The Gent model [29], which takes the strain-stiffening effect into consideration, is employed to represent the equilibrium and non-equilibrium elastic free energies, and Lagrange multipliers are used to impose the elastic deformation incompressibility of both ground networks and subnetworks. Finally, for model verification, we simulate the loading-unloading curves of VHB4905 at various thermo-mechanically and thermo-electric-mechanically coupling conditions and compare theoretical results with experimental data from Mehnert et al. [21] and Liao et al. [20].

The remainder of this paper is organized as follows: In Section 2, a continuum theoretical framework for thermo-electro-mechanically coupling problems is given, and constitutive equations, including the state and evolving equations, are derived. In Section 3, based on the proposed theoretical framework, specific constitutive equations are given and the thermo-electro-viscoelastic behaviors of VHB4905 are modeled. Finally, conclusions are given in Section 4.

## 2. Theoretical Framework

### 2.1. Kinematics

**Figure 1:**
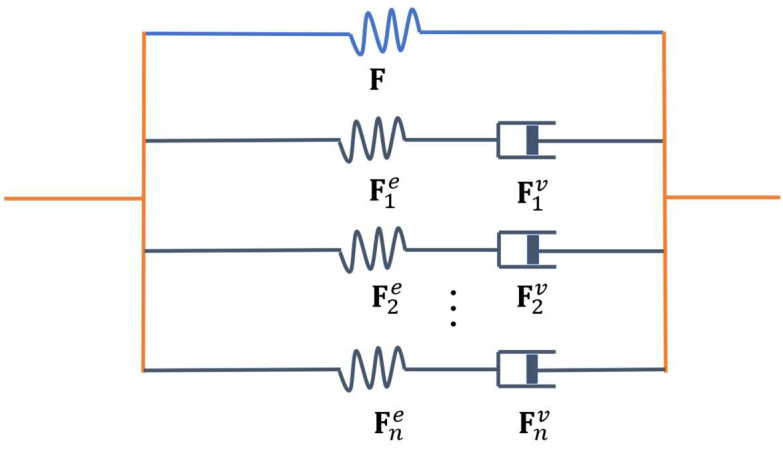
A one-dimensional analogy of the viscoelastic model for thermo-electro-mechanically coupling DEs at finite deformation. The networks of DEs are separated into a ground elastic network, with deformation gradient F, and n parallel viscous subnetworks, with an elastic deformation gradient $F_{i}^{e}$, and viscous deformation gradient $F_{i}^{v}$(1≤i≤n).

Consider a DE body $B_{0}$ bounded by the surface ${\partial B}_{0}$, which is defined in a fixed reference configuration and deforms into $B_{t}$ with the surface ${\partial B}_{t}$. Then the deformation gradient is given as follows:

(1) $$F={𝛻}_{R}x$$

where x=χ(X,t) is a function introduced to map an arbitrary material point X inside $B_{0}$ into a spatial point x inside $B_{t}$ and ${𝛻}_{R}$ is the gradient operator with respect to the coordinates X. To describe the viscoelasticity of DE at finite deformation, a one-dimensional analogy rheological model [22,25,30,31,32] is employed, as shown in Figure 1. Here, DE is assumed to comprise of a ground elastic network, with deformation gradient F, and n parallel viscous subnetworks, with elastic deformation gradient $F_{i}^{e}$ and viscous deformation gradient $F_{i}^{v}$, resulting in the following relation:

(2) $$F=F_{i}^{e}\cdot F_{i}^{v},\;\;i=1,...,n$$

The stress due to the deformation of the ground elastic network only depends on the F and cannot be relaxed, while the stress due to the elastic deformation $F_{i}^{e}$ can be relaxed with time because the viscous deformation gradient $F_{i}^{v}$ evolves with time.

Accordingly, the right Cauchy–Green deformation tensors, $C=F^{T}\cdot F$ and $C_{i}^{e}={\left(F_{i}^{e}\right)}^{T}\cdot F_{i}^{e}$ with the superscript T denoting the transpose operation, are used to measure, respectively, the deformation of the ground network and the elastic deformation of the subnetworks. 

### 2.2. Balance Laws and Entropy Inequality

Let d and $\rho^{c}$ denote the electrical displacement and the total charge density reckoned in the current configuration. Then, the Gauss law can be written as follows:

(3) $$\int_{\partial \Omega }d\cdot nda-\int_{\Omega }\rho^{c}dv=0$$

where n is the outward unit normal vector of the area element da on the surface ∂Ω with Ω denoting an arbitrary domain inside $B_{t}$, and dv is the volume element of Ω. The above equation can be equivalently written in the reference configuration as follows:

(4) $$\int_{\partial \Omega_{0}}D\cdot NdA-\int_{\Omega_{0}}\rho_{R}^{c}dV=0$$

where D and $\rho_{R}^{c}$ are, respectively, the electrical displacement and the total charge density reckoned in the reference configuration, N is the outward unit normal vector of the area element dA on the surface $\partial \Omega_{0}$ with $\Omega_{0}$ denoting an arbitrary domain inside $B_{0}$, and dV is the volume element of $\Omega_{0}$. The relations $D=jd\cdot {\left(F^{T}\right)}^{-1}$ and $\rho_{R}^{c}=j\rho^{c}$, with j as the determinate of F and the superscript ‘−1’ denoting the inverse of a tensor, can be obtained since $nda=j{\left(F^{T}\right)}^{-1}\cdot NdA$ and dv=jdV.

Using the divergence theorem, we can respectively write Equations (3) and (4) locally as follows:

(5) $$𝛻\cdot d=\rho^{c},\;\;\;\;\;\;\;\;\;\;\;{𝛻}_{R}\cdot D=\rho_{R}^{c}$$

where 𝛻 is the gradient operators with respect to the coordinates x, with $𝛻={𝛻}_{R}\cdot {\left(F^{T}\right)}^{-1}$. Furthermore, the electric field e, reckoned in the current configuration, and its counterpart E, reckoned in the reference configuration, are respectively defined by the following:

(6) $$e=-𝛻\Phi ,\;\;\;\;\;\;\;\;\;\;\;E=-{𝛻}_{R}\Phi$$

where Φ is the electric potential reckoned in both the current and reference configurations, and $E=F^{T}\cdot e$. 

Neglecting the inertial effects, the balance laws of force and moment are given in the current configuration as follows:

(7) $$\sigma \cdot 𝛻+b=0,\;\;\sigma =\sigma^{T}$$

where σ is the Cauchy stress tensor and b is the body force per unit volume in Ω, which can be given in the reference configuration as follows:

(8) $$P\cdot {𝛻}_{R}+b_{R}=0,\;\;P\cdot F^{T}=F\cdot P^{T}$$

where P is the first P-K stress tensor and $b_{R}$ is the body force per unit volume in $\Omega_{0}$, with the relations $P=j\sigma \cdot {\left(F^{T}\right)}^{-1}$ and $b_{R}=jb$.

Let ε and q denote, respectively, the internal energy density and the heat source per unit volume in Ω, and $j^{q}$ is the heat flux per unit area on ∂Ω. Then, the energy balance law in the current configuration is written as follows:

(9) $$\frac{d}{dt}\int_{\Omega }\varepsilon dv=\int_{\partial \Omega }\left(\sigma \cdot n\cdot \dot{\chi }-j^{q}\cdot n-\Phi \dot{d}\cdot n\right)da+\int_{\Omega }\left(b\cdot \dot{\chi }+q+\Phi {\dot{\rho }}^{c}\right)dv$$

whose corresponding form in the reference configuration is as follows:

(10) $$\frac{d}{dt}\int_{\Omega_{0}}\varepsilon_{R}dV=\int_{\partial \Omega_{0}}\left(P\cdot N\cdot \dot{\chi }-j_{R}^{q}\cdot N-\Phi \dot{D}\cdot N\right)dA+\int_{\Omega_{0}}\left(b_{R}\cdot \dot{\chi }+q_{R}+\Phi {\dot{\rho }}_{R}^{c}\right)dV$$

where $\frac{d}{dt}$ or, equivalently, a superposed dot represents the material time derivative $\varepsilon_{R}$ and $q_{R}$ denotes, respectively, the internal energy density and the heat source per unit volume in $\Omega_{0}$, and $j_{R}^{q}$ is the heat flux per unit area on $\partial \Omega_{0}$. The relations $\varepsilon_{R}=j\varepsilon$, $q_{R}=jq$, and $j_{R}^{q}=jj^{q}\cdot {\left(F^{T}\right)}^{-1}$ exist for the above two equations. The first and fourth terms on the right-hand side represent the mechanical work from the surface traction force and the body force, respectively; the second and fifth terms represent the thermal energy, respectively, from the heat flow across the surface and the heat source inside the body; and the third and sixth terms represent the electric work from the charge change on the surface and inside the body, respectively. From Equations (5)–(8) and using the divergence theorem, the local forms of Equations (9) and (10) can be respectively derived as follows:

(11) $$\dot{\varepsilon }=\sigma :𝛻\dot{\chi }-𝛻\cdot j^{q}+q+e\cdot \dot{d}$$

(12) $${\dot{\varepsilon }}_{R}=P:\dot{F}-{𝛻}_{R}\cdot j_{R}^{q}+q_{R}+E\cdot \dot{D}$$

Let η denote the entropy per unit volume in Ω and ϑ is the absolute temperature. The entropy inequality is written globally in the current configuration as follows:

(13) $$\frac{d}{dt}\int_{\Omega }\eta dv\geq \int_{\partial \Omega }\frac{-j^{q}\cdot n}{\vartheta }da+\int_{\Omega }\frac{q}{\vartheta }dv$$

which can also be written in the reference configuration as follows:

(14) $$\frac{d}{dt}\int_{\Omega_{0}}\eta_{R}dV\geq \int_{\partial \Omega_{0}}\frac{-j_{R}^{q}\cdot N}{\vartheta }dA+\int_{\Omega_{0}}\frac{q_{R}}{\vartheta }dV$$

where $\eta_{R}$ is the entropy per unit volume in $\Omega_{0}$ with the relation $\eta_{R}=j\eta$. Using the divergence theorem, the local forms of the above entropy inequalities are as follows:

(15) $$\dot{\eta }\geq -𝛻\cdot \frac{j^{q}}{\vartheta }+\frac{q}{\vartheta }$$

(16) $${\dot{\eta }}_{R}\geq -{𝛻}_{R}\cdot \frac{j_{R}^{q}}{\vartheta }+\frac{q_{R}}{\vartheta }$$

Introducing the Helmholtz free energy density φ=ε−ϑη and considering the energy balance (11), the inequality (15) becomes the following:

(17) $$\sigma :𝛻\dot{\chi }+e\cdot \dot{d}-\eta \dot{\vartheta }-\dot{\varphi }-\frac{1}{\vartheta }j^{q}\cdot 𝛻\vartheta \geq 0$$

Similarly, introducing the Helmholtz free energy density $\varphi_{R}=\varepsilon_{R}-\vartheta \eta_{R}$ with $\varphi_{R}=j\varphi$ and considering the energy balance (12), the inequality (16) becomes the following:

(18) $$P:\dot{F}+E\cdot \dot{D}-\eta_{R}\dot{\vartheta }-{\dot{\varphi }}_{R}-\frac{1}{\vartheta }j_{R}^{q}\cdot {𝛻}_{R}\vartheta \geq 0$$

which imposes the thermodynamic constraint on DEs. For convenience, we only employ the formulations given in the reference configuration in the following sections.

### 2.3. Constitutive Equations

In view of thermo-electro-viscoelastic effects of DEs, the Helmholtz free energy density $\varphi_{R}$ can be assumed as a function of variables $\left(C,C_{i}^{e},D,\vartheta \right)$, i.e.,

(19) $$\varphi_{R}=\varphi_{R}\left(C,C_{i}^{e},D,\vartheta \right)$$

so that its material time derivative can be written as follows:

(20) $${\dot{\varphi }}_{R}=\frac{\partial \varphi_{R}}{\partial C}:\dot{C}+\sum_{i=1}^{n}\frac{\partial \varphi_{R}}{\partial C_{i}^{e}}:{\dot{C}}_{i}^{e}+\frac{\partial \varphi_{R}}{\partial D}:\dot{D}+\frac{\partial \varphi_{R}}{\partial \vartheta }\dot{\vartheta }$$

The first and second terms on the right-hand side of the above equation can be rewritten as follows:

$$\frac{\partial \varphi_{R}}{\partial C}:\dot{C}=2F\cdot \frac{\partial \varphi_{R}}{\partial C}:\dot{F}$$

(21) $$\frac{\partial \varphi_{R}}{\partial C_{i}^{e}}:{\dot{C}}_{i}^{e}=\frac{\partial \varphi_{R}}{\partial C_{i}^{e}}:\frac{\partial C_{i}^{e}}{\partial F}:\dot{F}+\frac{\partial \varphi_{R}}{\partial C_{i}^{e}}:\frac{\partial C_{i}^{e}}{\partial F_{i}^{v}}:{\dot{F}}_{i}^{v}=2F_{i}^{e}\cdot \frac{\partial \varphi_{R}}{\partial C_{i}^{e}}\cdot {\left(F_{i}^{vT}\right)}^{-1}:\dot{F}-2C_{i}^{e}\cdot \frac{\partial \varphi_{R}}{\partial C_{i}^{e}}:D_{i}^{v}$$

with

(22) $$D_{i}^{v}=\frac{1}{2}\left(L_{i}^{v}+L_{i}^{vT}\right),\;\;L_{i}^{v}={\dot{F}}_{i}^{v}\cdot {\left(F_{i}^{v}\right)}^{-1}$$

Then, the substitution of Equations (20) and (21) into Equation (18) yields the following:

(23) $$\left(P-2F\cdot \frac{\partial \varphi_{R}}{\partial C}-2\sum_{i=1}^{n}F_{i}^{e}\cdot \frac{\partial \varphi_{R}}{\partial C_{i}^{e}}\cdot {\left(F_{i}^{vT}\right)}^{-1}\right):\dot{F}-\left(E-\frac{\partial \varphi_{R}}{\partial D}\right)\cdot \dot{D}-\left(\eta_{R}+\frac{\partial \varphi_{R}}{\partial \vartheta }\right)\dot{\vartheta }-\;\frac{1}{\vartheta }j_{R}^{q}\cdot {𝛻}_{R}\vartheta +\sum_{i=1}^{n}2C_{i}^{e}\cdot \frac{\partial \varphi_{R}}{\partial C_{i}^{e}}:D_{i}^{v}\geq 0$$

In the case that P, E, and $\eta_{R}$ are independent of $\dot{F}$, $\dot{D}$, and $\dot{\vartheta }$, the first three terms of the above inequality should vanish so that we have the following constitutive relations:

(24) $$P=2F\cdot \frac{\partial \varphi_{R}}{\partial C}+2\sum_{i=1}^{n}F_{i}^{e}\cdot \frac{\partial \varphi_{R}}{\partial C_{i}^{e}}\cdot {\left(F_{i}^{vT}\right)}^{-1}$$

(25) $$E=\frac{\partial \varphi_{R}}{\partial D}$$

(26) $$\eta_{R}=-\frac{\partial \varphi_{R}}{\partial \vartheta }$$

Thus, the inequality (23) reduces to the following:

(27) $$-\frac{1}{\vartheta }j_{R}^{q}\cdot {𝛻}_{R}\vartheta +\sum_{i=1}^{n}M_{i}^{neq}:D_{i}^{v}\geq 0$$

where $M_{i}^{neq}$ is the non-equilibrium Mandel stress tensor [33], defined as follows:

(28) $$M_{i}^{neq}=2C_{i}^{e}\cdot \frac{\partial \varphi_{R}}{\partial C_{i}^{e}}$$

More specifically, to satisfy the thermodynamic constraint given by inequality (27), the following constitutive equations are deduced:

(29) $$j_{R}^{q}=-Y\cdot {𝛻}_{R}\vartheta$$

(30) $$D_{i}^{v}=Q_{i}:M_{i}^{neq}$$

where Y is a second-order tensor and $Q_{i}$ a fourth-order tensor, both of which are positive-definite. Here, Equation (29) is the Fourier heat conduction law and Equation (30) is the rheological viscous flow rule [22,33].

### 2.4. Heat Conduction

Now, the first Gibbs relation can be obtained by substituting Equations (21), (24)–(26) and (28) into Equation (20), as follows:

(31) $${\dot{\varphi }}_{R}=P:\dot{F}+\sum_{i=1}^{n}M_{i}^{neq}:D_{i}^{v}+E\cdot \dot{D}-\eta_{R}\dot{\vartheta }$$

which can also be simultaneously transformed into the second Gibbs relation by using the relation $\varphi_{R}=\varepsilon_{R}-\vartheta \eta_{R}$, i.e.,

(32) $${\dot{\varepsilon }}_{R}=P:\dot{F}+\sum_{i=1}^{n}M_{i}^{neq}:D_{i}^{v}+E\cdot \dot{D}+\vartheta {\dot{\eta }}_{R}\;$$

Using the energy balance Equation (12), Equation (32) yields the following:

(33) $$\vartheta {\dot{\eta }}_{R}=-{𝛻}_{R}\cdot j_{R}^{q}+q_{R}+\sum_{i=1}^{n}M_{i}^{neq}:D_{i}^{v}$$

which indicates that the total entropy change includes the heat exchange of DE with its environment and its internal dissipation of heat due to viscous effects. From another perspective, it can also be found that the total entropy change includes the reversible entropy exchange (i.e., the right-hand side of the inequality (16)) and the irreversible entropy production (i.e., the left-hand side of the inequality (27)). Then, taking the derivative of Equation (26) with respect to time and substituting the result into Equation (33) gives the following:

(34) $$c\dot{\vartheta }=-{𝛻}_{R}\cdot j_{R}^{q}+q_{R}+\sum_{i=1}^{n}M_{i}^{neq}:D_{i}^{v}+\vartheta \frac{\partial P}{\partial \vartheta }:\dot{F}-\sum_{i=1}^{n}\vartheta \frac{\partial M_{i}^{neq}}{\partial \vartheta }:{\dot{D}}_{i}^{v}+\vartheta \frac{\partial E}{\partial \vartheta }\cdot \dot{D}$$

where c is the specific heat capacity, defined as $c=-\frac{\partial^{2}\varphi_{R}}{\partial \vartheta^{2}}$. This heat conduction equation explicitly indicates that temperature varies with heat exchange, viscous flow, the temperature-stress coupling effect, and the temperature-electricity coupling effect.

Up to now, we have obtained all the governing equations for the thermo-electro-viscoelastic process of DEs, including kinematic Equations (1), (2) and (22), balance Equations (5), (8) and (34), constitutive Equations (24)–(26) and (28)–(30), which can be solved under given boundary and initial conditions. In the next section, we will derive specific constitutive equations by giving specific Helmholtz free energy for DE and simulate viscoelastic phenomena of the DE polymer VHB4905 under various thermo-electro-mechanical loading conditions.

## 3. Special Cases

VHB4905 is one class of commercially available DEs, whose highly nonlinear stress-strain relations have been extensively investigated at various conditions [20,26,34]. In this section, specific constitutive equations for the coupling thermo-electro-viscoelastic behaviors of VHB4905 are first given and then used to simulate the loading-unloading curves under two different coupling conditions.

### 3.1. Specific Constitutive Equations

The Helmholtz free energy density is assumed to be additively decomposed, as follows:

(35) $$\varphi_{R}=\varphi_{R}^{eq}+\varphi_{R}^{neq}+\varphi_{R}^{E}+\varphi_{R}^{T}$$

where $\varphi_{R}^{eq}$ and $\varphi_{R}^{neq}$ are, respectively, the equilibrium and non-equilibrium Helmholtz free energy density resulting from the stretching of the ground network and subnetworks, $\varphi_{R}^{E}$ is the Helmholtz free energy density due to the interaction of quasi-static electric charges in VHB4905, and $\varphi_{R}^{T}$ is the Helmholtz free energy density due to temperature fluctuation.

Here, we adopt the Gent model [29] for $\varphi_{R}^{eq}$ and $\varphi_{R}^{neq}$ in consideration of the strain-stiffening effect that DEs may stiffen sharply when the network chain approaches its extension limit [35], as follows:

(36) $$\varphi_{R}^{eq}=-\frac{G^{eq}L}{2}\ln\left(\frac{L-trC+3}{L}\right)$$

(37) $$\varphi_{R}^{neq}=-\sum_{i=1}^{n}\frac{G_{i}^{neq}L_{i}}{2}\ln\left(\frac{L_{i}-trC_{i}^{e}+3}{L_{i}}\right)$$

where the symbol ‘tr’ denotes the trace of a tensor, $G^{eq}$ and $G_{i}^{neq}$ denote, respectively, the equilibrium modulus of the elastic ground network and the nonequilibrium modulus of the *i*th viscous subnetwork, and L and $L_{i}$ represent the extension limits of the elastic ground network and *i*th viscous subnetwork, respectively. 

For the sake of simplicity, an isotropic formulation of electrostatics in the current configuration is employed [36]:

(38) $$\frac{\varphi_{R}^{E}}{j}=\frac{1}{2\epsilon_{0}\epsilon_{r}}d\cdot d$$

whose corresponding form in the reference configuration can be obtained by using the relation $D=jd\cdot {\left(F^{T}\right)}^{-1}$, as follows:

(39) $$\varphi_{R}^{E}=\frac{1}{2j\epsilon_{0}\epsilon_{r}}\left(F^{T}\cdot F\right):\left(D⊗D\right)$$

where the symbol ⊗ denotes the dyadic product defining A⊗B as $A_{m}B_{k}$ for any vectors A (with $A_{m}\;as\;its\;component$) and B (with $B_{k}\;as\;its\;component$); and $\epsilon_{0}$ and $\epsilon_{r}$ are, respectively, the vacuum permittivity and the relative permittivity.

For the thermal part of the Helmholtz free energy density, we adopt the following [37]:

(40) $$\varphi_{R}^{T}=c_{0}\left(\vartheta -\vartheta_{0}-\vartheta \ln\frac{\vartheta }{\vartheta_{0}}\right)-c_{v}\frac{{\left(\vartheta -\vartheta_{0}\right)}^{2}}{2\vartheta_{0}}$$

which leads to a linear dependency of the specific heat capacity on temperature:

(41) $$c=c_{0}+c_{v}\frac{\vartheta }{\vartheta_{0}}$$

where $\vartheta_{0}$ is a reference temperature, and $c_{0}$ and $c_{v}$ are two coefficients of this linear dependency.

Substituting Equations (35)–(38) into (24), (25) and (28), we have

(42) $$P=\frac{G^{eq}L}{L-trC+3}F+\sum_{i=1}^{n}\frac{G_{i}^{neq}L_{i}}{L_{i}-trC_{i}^{e}+3}F_{i}^{e}\cdot {{(F}_{i}^{vT})}^{-1}+{\left(F^{T}\right)}^{-1}\;\cdot \left[\frac{1}{j\epsilon_{0}\epsilon_{r}}\left(C\cdot D\right)⊗D-\frac{1}{2}\left(\frac{1}{j\epsilon_{0}\epsilon_{r}}C\cdot D\cdot D\right)I\right]$$

(43) $$E=\frac{1}{j\epsilon_{0}\epsilon_{r}}C\cdot D$$

(44) $$M_{i}^{neq}=\frac{G_{i}^{neq}L_{i}}{L_{i}-trC_{i}^{e}+3}C_{i}^{e}$$

with I as the second-order unit tensor. 

Next, both the elastic deformations of the ground network and parallel subnetworks are considered to be incompressible [25], for which Lagrange multipliers Π and $\Pi_{i}$ are introduced to impose these constraint conditions and modify the free energy density as follows:

(45) $${\tilde{\varphi }}_{R}=\varphi_{R}-\Pi \left(j-1\right)-\sum_{i=1}^{n}\Pi_{i}\left(j_{i}^{e}-1\right)$$

where $j_{i}^{e}$ is the determinant of the elastic deformation gradient $F_{i}^{e}$. Replacing $\varphi_{R}$ with ${\tilde{\varphi }}_{R}$ in Equations (24) and (28), we can rewrite P and $M_{i}^{neq}$ as follows:

(46) $$P=\frac{G^{eq}L}{L-trC+3}F+\sum_{i=1}^{n}\frac{G_{i}^{neq}L_{i}}{L_{i}-trC_{i}^{e}+3}F_{i}^{e}\cdot {{(F}_{i}^{vT})}^{-1}+{\left(F^{T}\right)}^{-1}\cdot \left[E⊗D-\frac{1}{2}\left(E\cdot D\right)I\right]-\;j\Pi {\left(F^{T}\right)}^{-1}-\sum_{i=1}^{n}j_{i}^{e}\Pi_{i}{\left(F^{T}\right)}^{-1}$$

(47) $$M_{i}^{neq}=\frac{G_{i}^{neq}L_{i}}{L_{i}-trC_{i}^{e}+3}C_{i}^{e}-j_{i}^{e}\Pi_{i}I$$

where we have used the remaining unchanged relation (43). Furthermore, the Cauchy stress tensor σ can also be obtained by using the relations $\sigma =\frac{1}{j}P\cdot F^{T}$, $d=\epsilon_{0}\epsilon_{r}e$, $D=j{\left(F\right)}^{-1}\cdot d$, $E=F^{T}\cdot e$, and $j=j_{i}^{e}=1$ in Equation (46), as follows:

(48) $$\sigma =\frac{G^{eq}L}{L-trC+3}F\cdot F^{T}+\sum_{i=1}^{n}\frac{G_{i}^{neq}L_{i}}{L_{i}-trC_{i}^{e}+3}F_{i}^{e}\cdot F_{i}^{eT}+\epsilon_{0}\epsilon_{r}\left[e⊗e-\frac{1}{2}\left(e\cdot e\right)I\right]\;-\left(\Pi +\sum_{i=1}^{n}\Pi_{i}\right)I$$

Employing $j=j_{i}^{e}=1$ and the multiplicative decomposition of F in Equation (2), the viscous incompressibility, $trD_{i}^{v}=0$ or $j_{i}^{v}=1$ with $j_{i}^{v}$ as the determinant of $F_{i}^{v}$, can be deduced, from which the fourth-order tensor $Q_{i}$ in Equation (30) can be taken as follows [25]:

(49) $$Q_{i}=\frac{1}{2v_{i}}\left(I_{4}-\frac{1}{3}I⊗I\right)$$

where $I_{4}$ is the fourth-order unit tensor and $v_{i}$ the viscosity of the ith subnetwork. Then, the relaxation time for the ith subnetwork can be defined as $\tau_{i}=\frac{v_{i}}{G_{i}^{neq}}$ [31].

### 3.2. Thermo-Viscoelastic Coupling

In the thermo-viscoelastic experiments of Mehnert et al. [21], VHB4905 samples with the dimensions 130 mm×10 mm×0.5 mm are first heated at different temperatures in the thermal chamber for about 10 min, which has proven to be sufficient for the samples to induce the temperature of the chamber, and then mounted onto the machine *Inspekt 5 kN* for multi-step relaxation tests and cyclic loading-unloading tests.

During heating, we assume that the deformation of the samples can be neglected due to the relatively small thermal expansion coefficient and the free boundary conditions. Substituting Equations (41) and (29) into Equation (34) and neglecting all the heat sources, the heat conduction equation reduces to the following:

(50) $$\left(c_{0}+c_{v}\frac{\vartheta }{\vartheta_{0}}\right)\dot{\vartheta }={𝛻}_{R}\cdot \left(Y\cdot {𝛻}_{R}\vartheta \right)$$

where we consider the isotropic Fourier heat conduction so that $Y=\left(\kappa_{0}+\kappa_{v}\frac{\vartheta }{\vartheta_{0}}\right)I$ with $\kappa_{0}$ and $\kappa_{v}$ are two coefficients of the linear temperature-dependent conductivity [38]. The Neumann boundary condition is used to describe the convective heat transfer between the sample and the air in the thermal chamber, given as follows:

(51) $$j_{R}\cdot n=h\left(\vartheta_{s}-\vartheta_{t}\right)$$

where h is the convective heat transfer coefficient, $\vartheta_{s}$ and $\vartheta_{t}$ are, respectively, the temperature of the sample surface and the temperature of air in the thermal chamber. Here, the room temperature (296 K) is taken as the reference temperature (i.e., $\vartheta_{0}=296\;K$) and the temperature of air in the thermal chamber is set as $\vartheta_{t}=$ 353 K. The initial condition for the temperature of the sample is assumed to be as follows:

(52) $${\left.\vartheta \right|}_{t=0}=296\;K$$

The material parameters, $c_{0},c_{v},\kappa_{0},$ and $\kappa_{v}$, used in the calculation are given in Table 1, based on Dippel et al. [38] for natural rubber, which resembles VHB4905 in molecular structure. Furthermore, considering that the convective heat transfer coefficient for forced gas convection is about $20\sim 300\;W/\left(m^{2}\cdot K\right)$, $h=20\;W/\left(m^{2}\cdot K\right)$ is chosen, as given in Table 1, for the convective heat transfer between the sample and the air in the thermal chamber so that we can roughly estimate the maximum time for reaching the steady state of heat conduction.

**Figure 2:**
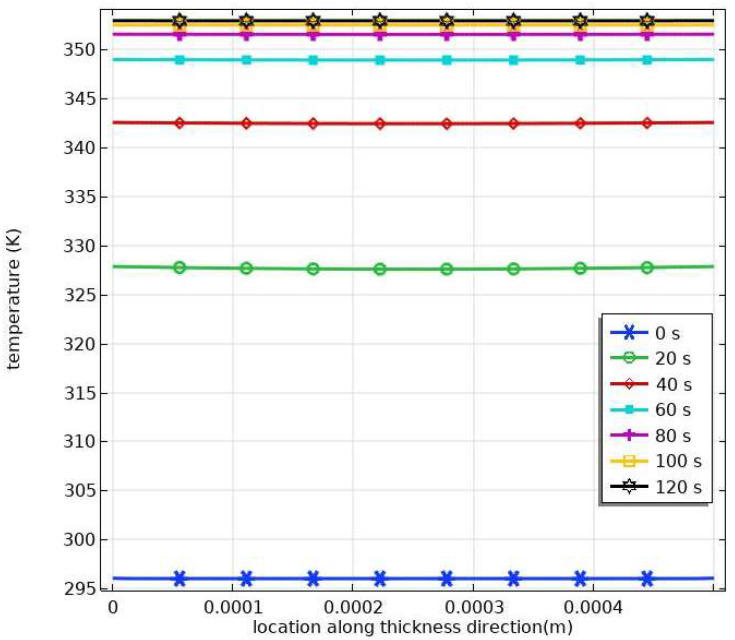
Temperature distribution along the thickness direction at different times during heating.

The heat conduction inside VHB4905 samples can be simplified as a one-dimensional problem since the dimension in the thickness direction is much smaller than the other two lateral dimensions, which results in negligible convective heat transfer on the lateral surfaces. By solving Equation (50) with the boundary condition (51) and the initial condition (52) through the commercial software COMSOL Multiphysic 5.3, the temperature distribution along the thickness direction during heating is obtained and shown in Figure 2. It can be seen that the temperature along thickness direction is nearly uniform and increases with time until it reaches 353 K (the temperature in the thermal chamber). This is because the great heat conductivity of VHB4905 leads to rapid heat transfer across a small distance between neighboring surfaces (0.5 mm), and forced gas convection between the sample and the air in the thermal chamber results in a steady temperature distribution after about 100 s. The numerical results reveal that 10 min is sufficient for VHB4905 samples to induce the temperature of the thermal chamber.

Next, the corresponding deformation gradient in the mechanical tests can be written as follows:

(53) $$\begin{array}{c} F=\left[\begin{array}{ccc} \lambda_{1} & 0 & 0\\ 0 & \lambda_{2} & 0\\ 0 & 0 & \lambda_{3} \end{array}\right],\;\;\;F_{i}^{e}=\left[\begin{array}{ccc} \lambda_{i1}^{e} & 0 & 0\\ 0 & \lambda_{i2}^{e} & 0\\ 0 & 0 & \lambda_{i3}^{e} \end{array}\right],\;\;\;F_{i}^{v}=\left[\begin{array}{ccc} \lambda_{i1}^{v} & 0 & 0\\ 0 & \lambda_{i2}^{v} & 0\\ 0 & 0 & \lambda_{i3}^{v} \end{array}\right]\\ D_{i}^{v}=\left[\begin{array}{ccc} {\dot{\lambda }}_{i1}^{v}/\lambda_{i1}^{v} & 0 & 0\\ 0 & {\dot{\lambda }}_{i2}^{v}/\lambda_{i2}^{v} & 0\\ 0 & 0 & {\dot{\lambda }}_{i3}^{v}/\lambda_{i3}^{v} \end{array}\right] \end{array}$$

Here $\lambda_{1}$, $\lambda_{2}$, and $\lambda_{3}$ are the principle stretches of the deformation gradient F; $\lambda_{i1}^{e}$, $\lambda_{i2}^{e}$, and $\lambda_{i3}^{e}$ are the principle stretches of the elastic deformation gradient $F_{i}^{e}$; and $\lambda_{i1}^{v}$, $\lambda_{i2}^{v}$, and $\lambda_{i3}^{v}$ are the principle stretches of the viscous deformation gradient $F_{i}^{v}$. In consideration of the equal lateral stretches during the uniaxial tensile test and the incompressible deformation of VHB4905 samples ($j=j_{i}^{e}=j_{i}^{v}=1$), we have the following:

$$\lambda_{1}=\lambda ,\;\;\lambda_{2}=\lambda_{3}={\left(\lambda \right)}^{-\frac{1}{2}}$$

$$\lambda_{1}=\lambda_{i1}^{e}\lambda_{i1}^{v}=\lambda ,\;\lambda_{i2}^{e}\lambda_{i2}^{v}=\lambda_{i3}^{e}\lambda_{i3}^{v}={\left(\lambda \right)}^{-\frac{1}{2}}$$

(54) $$\lambda_{i1}^{e}=\lambda_{i}^{e},\;\lambda_{i2}^{e}=\lambda_{i3}^{e}={\left(\lambda_{i}^{e}\right)}^{-\frac{1}{2}},\lambda_{i1}^{v}=\lambda_{i}^{v},\lambda_{i2}^{v}=\lambda_{i3}^{v}={\left(\lambda_{i}^{v}\right)}^{-\frac{1}{2}}$$

with λ, $\lambda_{i}^{e}$, and $\lambda_{i}^{v}$, respectively, as the principle stretches of the deformation gradients F, $F_{i}^{e}$, and $F_{i}^{v}$ along the tensile direction. 

Let $P_{1}$, $P_{2}$, and $P_{3}$ denote the components of P along three principal directions, respectively. Substituting Equations (53) and (54) into Equation (46) and then discarding the electric terms, we have the following:

$$P_{1}=\frac{G^{eq}L\lambda }{L-2\lambda^{-1}-\lambda^{2}+3}+\sum_{i=1}^{n}\frac{G_{i}^{neq}L_{i}{\lambda (\lambda_{i}^{v})}^{-2}}{L_{i}-2\lambda^{-1}\lambda_{i}^{v}-\lambda^{2}{\left(\lambda_{i}^{v}\right)}^{-2}+3}-\frac{\Pi +\sum_{i=1}^{n}\Pi_{i}}{\lambda }$$

(55) $$P_{2}=P_{3}=\frac{G^{eq}L\lambda^{-\frac{1}{2}}}{L-2\lambda^{-1}-\lambda^{2}+3}+\sum_{i=1}^{n}\frac{G_{i}^{neq}L_{i}\lambda^{-\frac{1}{2}}\lambda_{i}^{v}}{L_{i}-2\lambda^{-1}\lambda_{i}^{v}-\lambda^{2}{\left(\lambda_{i}^{v}\right)}^{-2}+3}-\left(\Pi +\sum_{i=1}^{n}\Pi_{i}\right)\lambda^{\frac{1}{2}}$$

According to the boundary condition, $P_{2}=P_{3}=0$ for the uniaxial loading-unloading tests, we can further obtain the following:

(56) $$\Pi +\sum_{i=1}^{n}\Pi_{i}=\frac{G^{eq}L\lambda^{-1}}{L-2\lambda^{-1}-\lambda^{2}+3}+\sum_{i=1}^{n}\frac{G_{i}^{neq}L_{i}\lambda^{-1}\lambda_{i}^{v}}{L_{i}-2\lambda^{-1}\lambda_{i}^{v}-\lambda^{2}{\left(\lambda_{i}^{v}\right)}^{-2}+3}$$

and

(57) $$P_{1}=\frac{G^{eq}L(\lambda -\lambda^{-2})}{L-2\lambda^{-1}-\lambda^{2}+3}+\sum_{i=1}^{n}\frac{G_{i}^{neq}L_{i}\left[{\lambda (\lambda_{i}^{v})}^{-2}-\lambda^{-2}\lambda_{i}^{v}\right]}{L_{i}-2\lambda^{-1}\lambda_{i}^{v}-\lambda^{2}{\left(\lambda_{i}^{v}\right)}^{-2}+3}$$

Here, the incompressibilities of the ground network and the parallel subnetworks have an effect on the stress, and Lagrange multipliers are eliminated according to the boundary conditions. 

Let $M_{i1}^{neq}$, $M_{i2}^{neq}$, and $M_{i3}^{neq}$ denote the components of $M_{i}^{neq}$ along three principal directions, respectively. Similarly, substituting Equations (53) and (54) into Equation (47), we have the following:

(58) $$\begin{array}{c} M_{i1}^{neq}=\frac{G_{i}^{neq}L_{i}\lambda^{2}{\left(\lambda_{i}^{v}\right)}^{-2}}{L_{i}-2\lambda^{-1}\lambda_{i}^{v}-\lambda^{2}{\left(\lambda_{i}^{v}\right)}^{-2}+3}-\Pi_{i}\\ M_{i2}^{neq}=M_{i3}^{neq}=\frac{G_{i}^{neq}L_{i}\lambda^{-1}\lambda_{i}^{v}}{L_{i}-2\lambda^{-1}\lambda_{i}^{v}-\lambda^{2}{\left(\lambda_{i}^{v}\right)}^{-2}+3}-\Pi_{i} \end{array}$$

Then, substitution of Equations (58), (53) and (49) into Equation (30) yields the following:

(59) $$\frac{{\dot{\lambda }}_{i}^{v}}{\lambda_{i}^{v}}=\frac{L_{i}}{3\tau_{i}\left[L_{i}-2\lambda^{-1}\lambda_{i}^{v}-\lambda^{2}{\left(\lambda_{i}^{v}\right)}^{-2}+3\right]}\left[\lambda^{2}{\left(\lambda_{i}^{v}\right)}^{-2}-\lambda^{-1}\lambda_{i}^{v}\right]$$

which describes the viscous flow in VHB4905 subjected to uniaxial tension. It is worth noting that the elastic incompressibility of the subnetworks has no influence on the viscous flow since only the deviatoric stress, excluding the hydrostatic pressure $\Pi_{i}$, promotes the viscous flow.

Under the initial conditions, ${\left.P_{1}\right|}_{t=0}=0$, ${\left.\lambda \right|}_{t=0}={\left.\lambda_{i}^{v}\right|}_{t=0}=1$, the coupled Equations (57) and (59) can be simultaneously solved to predict the mechanical behaviors under the given material parameters. To determine the material parameters, the least square method is used to fit Equations (57) and (59) with the experimental data of Mehnert et al. [21], who performed multi-step relaxation tests for identifying the elastic responses at different stretches and cyclic loading-unloading tests for acquiring the viscous information at different stretching rates. In each step of a multi-step relaxation test with eight consecutive steps, the sample is stretched quickly with an increased 25% deformation and then held at this deformation state for thirty minutes (the viscous stress is considered to be relaxed almost completely after this period), for which Equations (57) and (59) can be reduced to the equilibrium equation:

(60) $$P_{1}=\frac{G^{eq}L(\lambda -\lambda^{-2})}{L-2\lambda^{-1}-\lambda^{2}+3}$$

**Figure 3:**
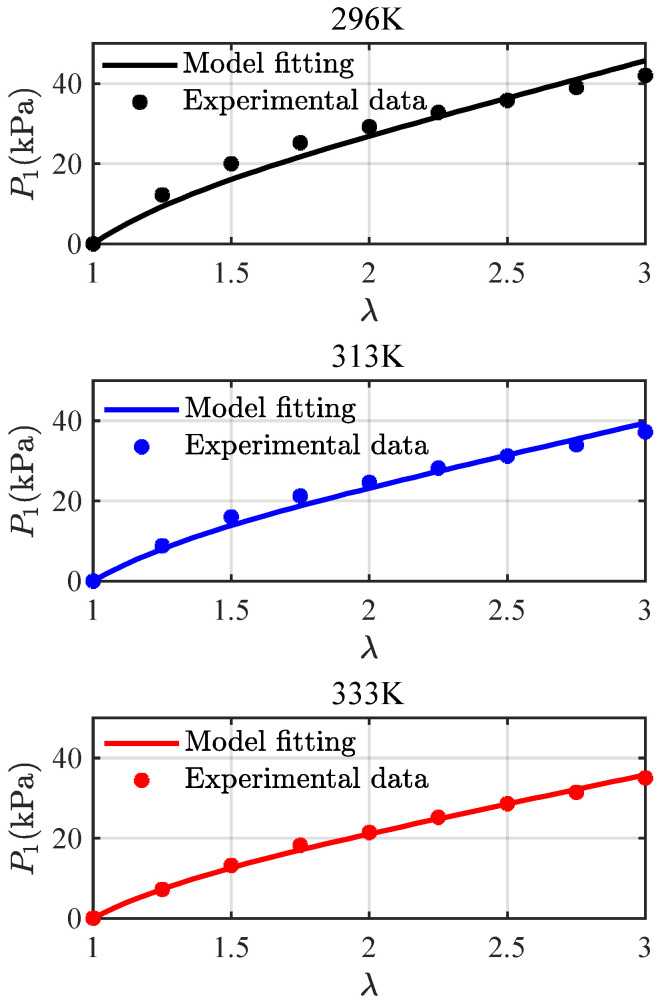
A comparison of the equilibrium stress-stretch relations at 296 K, 313 K, and 333 K from the model fitting and the experimental data [21].

**Figure 4:**
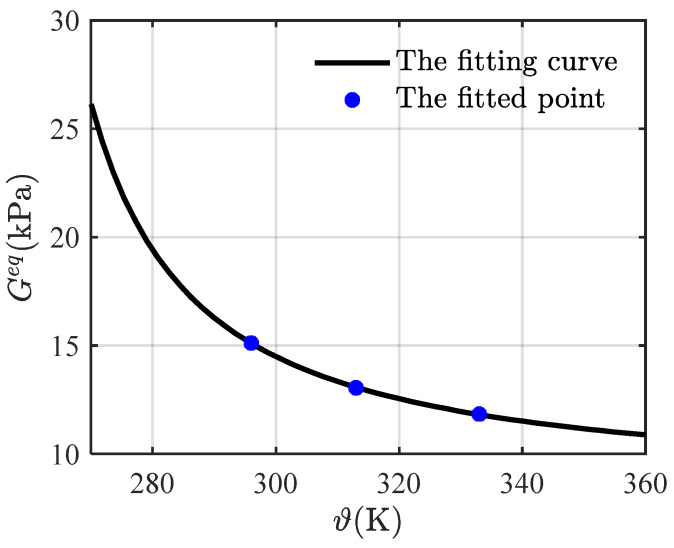
The fitting curve of the equilibrium modulus versus temperature.

Here, we take L=155, according to Kollosche et al. [39], and fit Equation (60) with the multi-step relaxation tests [21] to obtain $G^{eq}$ = 15.12 kPa, 13.05 kPa, and 11.84 kPa for the temperatures 296 K, 313 K, and 333 K, respectively. Figure 3 gives a comparison of the equilibrium stress-stretch relations, respectively, from model fitting and experimental data. The experimental data are in good agreement with the fitting curves, indicating that Equation (60) is capable of predicting the equilibrium response of VHB4905. Three discrete points (296, 15.12), (313, 13.05), and (333, 11.84) are further used to fit, as shown in Figure 4, the following exponential function:

(61) $$G^{eq}\left(\vartheta \right)=aexp\left(\frac{b\left(\vartheta -\vartheta_{g}\right)}{\vartheta -\vartheta_{g}-c}\right)$$

with a=0.13 kPa,b=4.16, and c=8.06 K. Here, $\vartheta_{g}$ (233 K) is the glass transition temperature of VHB4905. This dependence of the equilibrium modulus on temperature is of the Williams–Landel–Ferry (WLF) type [40], and indicates that increasing temperature would soften VHB4905. This function can be used to calculate $G^{eq}=25.56\;kPa$ for 273 K. 

**Figure 5:**
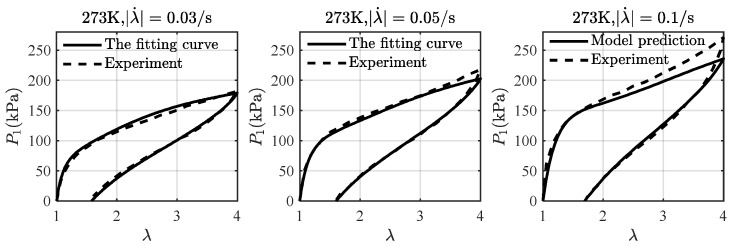
A comparison of the loading-unloading curves at 273 K. The experimental data of the stretching rate $\left|\dot{\lambda }\right|=0.03/s$ and $\left|\dot{\lambda }\right|=0.05/s$ are used for the model fitting, and $\left|\dot{\lambda }\right|=0.1/s$ is used for the model validation. The experimental data are taken from Liao et al. [20].

Figure 5 gives a comparison of the experimental data with the model fitting and the model prediction at different stretching rates and a fixed temperature of 273 K. We have found that n=3 results in well-converged results as well as physically realistic parameter values. With $G^{eq}=25.56\;kPa$ and $L=L_{i}=155$ (i=1, 2, 3) determined, the non-equilibrium modulus $G_{i}^{neq}\;(38.44\;kPa,\;94.23\;kPa,\;94.96\;kPa)$ and the relaxation time $\tau_{i}\;(413.32\;s,\;5.43\;s,\;1.65\;s)$ are obtained by fitting Equations (57) and (59) with the experimental data at stretching rates $\left|\dot{\lambda }\right|=0.03/s$ and $\left|\dot{\lambda }\right|=0.05/s$ in Figure 5. Then, the model prediction of $\left|\dot{\lambda }\right|=0.1/s$ by using all determined parameters agrees well with the experimental data, and each loading-unloading cycle shows typical viscoelastic behavior, which validates the employed viscoelastic model.

**Figure 6:**
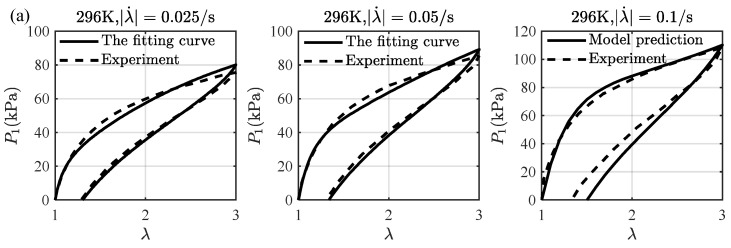

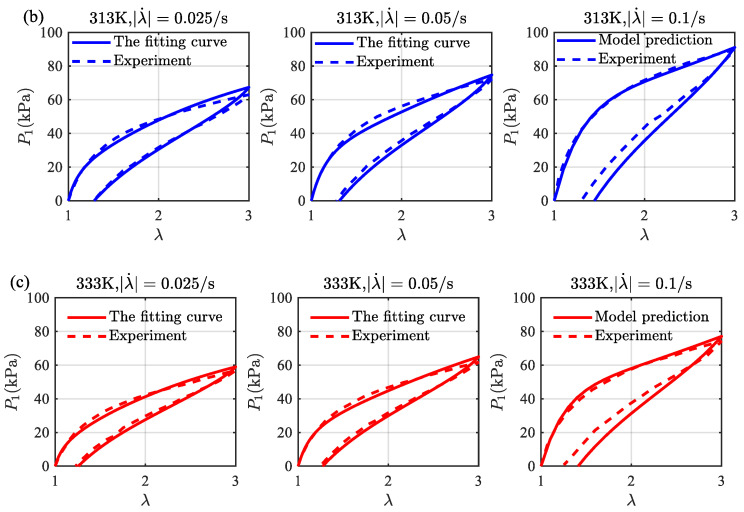
A comparison of the loading-unloading curves at (**a**) 296 K, (**b**) 313 K, and (**c**) 333 K. The experimental data of the stretching rate $\left|\dot{\lambda }\right|=0.025/s$ and $\left|\dot{\lambda }\right|=0.05/s$ are used for the model fitting, and $\left|\dot{\lambda }\right|=0.1/s$ is used for the model validation. The experimental data are taken from Mehnert et al. [21].

Figure 6 also presents a comparison of the experimental data with the model fitting and the model prediction at different stretching rates and temperatures. Here, we assume that the relaxation times and the extension limits are temperature-independent, so that only the dependence of the non-equilibrium moduli on temperature is obtained via the model fitting. The experimental data at the stretching rates $\left|\dot{\lambda }\right|=0.025/s$ and $\left|\dot{\lambda }\right|=0.05/s$ are used to determine the non-equilibrium moduli, and $\left|\dot{\lambda }\right|=0.1/s$ is used to validate the model fitting. It can be seen that the results from the model fitting and the model prediction are close to the experimental data, and the viscoelastic effect at the higher temperature is more significant, indicating that the strategy is reasonable. We assume that the dependence of the non-equilibrium moduli on temperature is also a WLF type like Equation (61) and fit this type with the obtained four discrete points, yielding the following fitting functions:

(62) $$\begin{array}{c} G_{1}^{neq}\left(\vartheta \right)=5.54\times 10^{-2}\exp\left(\frac{4.74\left(\vartheta -233\right)}{\vartheta -233-11.04}\right)\\ G_{2}^{neq}\left(\vartheta \right)=6.79\times 10^{-3}\exp\left(\frac{6.87\left(\vartheta -233\right)}{\vartheta -233-11.17}\right)\\ G_{3}^{neq}\left(\vartheta \right)=7.89\times 10^{-3}\exp\left(\frac{7.21\left(\vartheta -233\right)}{\vartheta -233-9.34}\right) \end{array}$$

**Figure 7:**
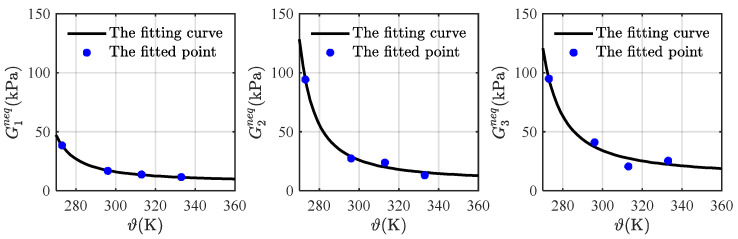
The fitting curves of the non-equilibrium moduli versus temperature.

The fitting curves of the non-equilibrium modulus versus temperature and the corresponding fitted points are depicted in Figure 7, where the fitting curves match roughly with these points. With these assumptions and relations, all parameters at 353 K (see Table 2) are obtained and substituted into Equations (57) and (59) for predicting the loading-unloading curves at different stretching rates. 

**Figure 8:**
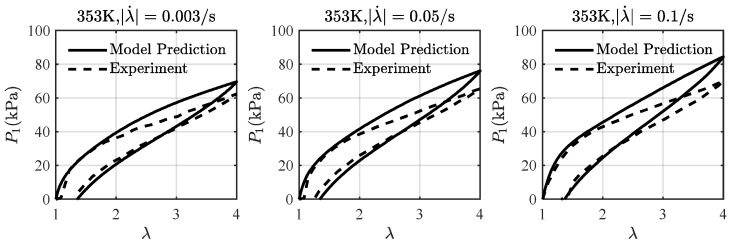
A comparison of the loading-unloading curves from the model prediction and the experimental data at $\left|\dot{\lambda }\right|=0.025,\;0.05,\frac{0.1}{s},$ and 353 K. The experimental data are taken from Liao et al. [20].

Figure 8 shows a comparison of the loading-unloading curves from the model prediction and the experimental data at $\left|\dot{\lambda }\right|=0.025,\;0.05,\frac{0.1}{s},$ and 353 K. It can be seen that the predicted loading-unloading curves from the present model match reasonably well with the experimental results, and the peak stress of each curve increases with the stretching rate. The comparison indicates that the present model and its fitting parameters are able to describe the viscoelastic effect of VHB4905 at different stretching rates and temperatures.

### 3.3. Thermo-Electro-Viscoelastic Coupling

In the thermo-electro-viscoelastic experiments of VHB4905 by Mehnert et al. [21], material samples with the dimensions 70 mm×100 mm×0.5 mm, coated with carbon conductive grease, acting as compliant electrodes, on its two largest surfaces, are first heated for fifteen minutes to reach a target temperature in the closed thermal chamber. Then, an electric field is applied on the two largest surfaces of the heated samples. Finally, the loading-unloading tests on the samples with different electric fields are conducted.

Let E denote the electric field along thickness direction reckoned in the reference configuration. Substitution of Equation (53) into Equation (46) yields the following:

$$P_{1}=\frac{G^{eq}L\lambda_{1}}{L-\lambda_{1}^{2}-\lambda_{2}^{2}-\lambda_{3}^{2}+3}+\sum_{i=1}^{n}\frac{G_{i}^{neq}L_{i}\lambda_{1}{\left(\lambda_{i1}^{v}\right)}^{-2}}{L_{i}-\lambda_{1}^{2}{\left(\lambda_{i1}^{v}\right)}^{-2}-\lambda_{2}^{2}{\left(\lambda_{i2}^{v}\right)}^{-2}-\lambda_{3}^{2}{\left(\lambda_{i3}^{v}\right)}^{-2}+3}-\frac{\Pi +\sum_{i=1}^{n}\Pi_{i}}{\lambda_{1}}-\epsilon_{0}\epsilon_{r}\frac{E^{2}}{2\lambda_{1}\lambda_{3}^{2}}$$

$$P_{2}=\frac{G^{eq}L\lambda_{2}}{L-\lambda_{1}^{2}-\lambda_{2}^{2}-\lambda_{3}^{2}+3}+\sum_{i=1}^{n}\frac{G_{i}^{neq}L_{i}\lambda_{2}{\left(\lambda_{i2}^{v}\right)}^{-2}}{L_{i}-\lambda_{1}^{2}{\left(\lambda_{i1}^{v}\right)}^{-2}-\lambda_{2}^{2}{\left(\lambda_{i2}^{v}\right)}^{-2}-\lambda_{3}^{2}{\left(\lambda_{i3}^{v}\right)}^{-2}+3}-\frac{\Pi +\sum_{i=1}^{n}\Pi_{i}}{\lambda_{2}}-\epsilon_{0}\epsilon_{r}\frac{E^{2}}{2\lambda_{2}\lambda_{3}^{2}}$$

(63) $$P_{3}=\frac{G^{eq}L\lambda_{3}}{L-\lambda_{1}^{2}-\lambda_{2}^{2}-\lambda_{3}^{2}+3}+\sum_{i=1}^{n}\frac{G_{i}^{neq}L_{i}\lambda_{3}{\left(\lambda_{i3}^{v}\right)}^{-2}}{L_{i}-\lambda_{1}^{2}{\left(\lambda_{i1}^{v}\right)}^{-2}-\lambda_{2}^{2}{\left(\lambda_{i2}^{v}\right)}^{-2}-\lambda_{3}^{2}{\left(\lambda_{i3}^{v}\right)}^{-2}+3}-\frac{\Pi +\sum_{i=1}^{n}\Pi_{i}}{\lambda_{3}}+\epsilon_{0}\epsilon_{r}\frac{E^{2}}{2\lambda_{3}^{2}}$$

From the boundary condition $P_{3}=0$, we can obtain the following:

(64) $$\Pi +\sum_{i=1}^{n}\Pi_{i}=\frac{G^{eq}L\lambda_{3}^{2}}{L-\lambda_{1}^{2}-\lambda_{2}^{2}-\lambda_{3}^{2}+3}+\sum_{i=1}^{n}\frac{G_{i}^{neq}L_{i}\lambda_{3}^{2}{\left(\lambda_{i3}^{v}\right)}^{-2}}{L_{i}-\lambda_{1}^{2}{\left(\lambda_{i1}^{v}\right)}^{-2}-\lambda_{2}^{2}{\left(\lambda_{i2}^{v}\right)}^{-2}-\lambda_{3}^{2}{\left(\lambda_{i3}^{v}\right)}^{-2}+3}-{\epsilon_{0}\epsilon }_{r}\frac{E^{2}}{2\lambda_{3}^{2}}$$

Further considering the incompressibility constraints $\lambda_{1}\lambda_{2}\lambda_{3}=\lambda_{i1}^{v}\lambda_{i2}^{v}\lambda_{i3}^{v}=1,$ and the boundary conditions $P_{2}=0$, we obtain the following:

$$P_{1}=\frac{G^{eq}L\left(\lambda_{1}-\lambda_{1}^{-3}\lambda_{2}^{-2}\right)}{L-\lambda_{1}^{2}-\lambda_{2}^{2}-\lambda_{1}^{-2}\lambda_{2}^{-2}+3}+\sum_{i=1}^{n}\frac{G_{i}^{neq}L_{i}\left[\lambda_{1}{\left(\lambda_{i1}^{v}\right)}^{-2}-\lambda_{1}^{-3}\lambda_{2}^{-2}{\left(\lambda_{i1}^{v}\lambda_{i2}^{v}\right)}^{2}\right]}{L_{i}-\lambda_{1}^{2}{\left(\lambda_{i1}^{v}\right)}^{-2}-\lambda_{2}^{2}{\left(\lambda_{i2}^{v}\right)}^{-2}-\lambda_{1}^{-2}\lambda_{2}^{-2}{\left(\lambda_{i1}^{v}\lambda_{i2}^{v}\right)}^{2}+3}\;-\epsilon_{0}\epsilon_{r}{\lambda_{2}^{2}\lambda_{1}E}^{2}$$

(65) $$P_{2}=\frac{G^{eq}L\left(\lambda_{2}-\lambda_{1}^{-2}\lambda_{2}^{-3}\right)}{L-\lambda_{1}^{2}-\lambda_{2}^{2}-\lambda_{1}^{-2}\lambda_{2}^{-2}+3}+\sum_{i=1}^{n}\frac{G_{i}^{neq}L_{i}\left[\lambda_{2}{\left(\lambda_{i2}^{v}\right)}^{-2}-\lambda_{1}^{-2}\lambda_{2}^{-3}{\left(\lambda_{i1}^{v}\lambda_{i2}^{v}\right)}^{2}\right]}{L_{i}-\lambda_{1}^{2}{\left(\lambda_{i1}^{v}\right)}^{-2}-\lambda_{2}^{2}{\left(\lambda_{i2}^{v}\right)}^{-2}-\lambda_{1}^{-2}\lambda_{2}^{-2}{\left(\lambda_{i1}^{v}\lambda_{i2}^{v}\right)}^{2}+3}-\epsilon_{0}\epsilon_{r}\lambda_{2}\lambda_{1}^{2}E^{2}=0$$

Next, substituting Equation (53) into Equation (47), we have the following:

$$M_{i1}^{neq}=\frac{G_{i}^{neq}L_{i}\lambda_{1}^{2}{\left(\lambda_{i1}^{v}\right)}^{-2}}{L_{i}-\lambda_{1}^{2}{\left(\lambda_{i1}^{v}\right)}^{-2}-\lambda_{2}^{2}{\left(\lambda_{i2}^{v}\right)}^{-2}-\lambda_{3}^{2}{\left(\lambda_{i3}^{v}\right)}^{-2}+3}-\Pi_{i}$$

$$M_{i2}^{neq}=\frac{G_{i}^{neq}L_{i}\lambda_{2}^{2}{\left(\lambda_{i2}^{v}\right)}^{-2}}{L_{i}-\lambda_{1}^{2}{\left(\lambda_{i1}^{v}\right)}^{-2}-\lambda_{2}^{2}{\left(\lambda_{i2}^{v}\right)}^{-2}-\lambda_{3}^{2}{\left(\lambda_{i3}^{v}\right)}^{-2}+3}-\Pi_{i}$$

(66) $$M_{i3}^{neq}=\frac{G_{i}^{neq}L_{i}\lambda_{3}^{2}{\left(\lambda_{i3}^{v}\right)}^{-2}}{L_{i}-\lambda_{1}^{2}{\left(\lambda_{i1}^{v}\right)}^{-2}-\lambda_{2}^{2}{\left(\lambda_{i2}^{v}\right)}^{-2}-\lambda_{3}^{2}{\left(\lambda_{i3}^{v}\right)}^{-2}+3}-\Pi_{i}$$

Substituting Equations (49), (53) and (66) into Equation (30) and using the incompressibility constraints $\lambda_{1}\lambda_{2}\lambda_{3}=\lambda_{i1}^{v}\lambda_{i2}^{v}\lambda_{i3}^{v}=1$, we can obtain the viscous flow rule:

$$\frac{{\dot{\lambda }}_{i1}^{v}}{\lambda_{i1}^{v}}=\frac{L_{i}}{3\tau_{i}\left[L_{i}-\lambda_{1}^{2}{\left(\lambda_{i1}^{v}\right)}^{-2}-\lambda_{2}^{2}{\left(\lambda_{i2}^{v}\right)}^{-2}-\lambda_{1}^{-2}\lambda_{2}^{-2}{\left(\lambda_{i1}^{v}\lambda_{i2}^{v}\right)}^{2}+3\right]}[\lambda_{1}^{2}{\left(\lambda_{i1}^{v}\right)}^{-2}\;-\frac{\lambda_{2}^{2}{\left(\lambda_{i2}^{v}\right)}^{-2}+{\lambda_{1}^{-2}\lambda }_{2}^{-2}{\left(\lambda_{i1}^{v}\lambda_{i2}^{v}\right)}^{2}}{2}]$$

(67) $$\frac{{\dot{\lambda }}_{i2}^{v}}{\lambda_{i2}^{v}}=\frac{L_{i}}{3\tau_{i}\left[L_{i}-\lambda_{1}^{2}{\left(\lambda_{i1}^{v}\right)}^{-2}-\lambda_{2}^{2}{\left(\lambda_{i2}^{v}\right)}^{-2}-\lambda_{1}^{-2}\lambda_{2}^{-2}{\left(\lambda_{i1}^{v}\lambda_{i2}^{v}\right)}^{2}+3\right]}[\lambda_{2}^{2}{\left(\lambda_{i2}^{v}\right)}^{-2}\;-\frac{\lambda_{1}^{2}{\left(\lambda_{i1}^{v}\right)}^{-2}+{\lambda_{1}^{-2}\lambda }_{2}^{-2}{\left(\lambda_{i1}^{v}\lambda_{i2}^{v}\right)}^{2}}{2}]$$

Here, it is worth noting that the hydrostatic pressure $\Pi_{i}$ is eliminated by the mathematical operation since only the deviatoric stress is considered to promote the viscous flow in Equation (49).

Furthermore, an electric field prior to the mechanical test leads to an extension of the sample in the transverse direction, so we have $\lambda_{3}=\lambda_{pre}$ and $\lambda_{1}=\lambda_{2}=\frac{1}{\sqrt{\lambda_{pre}}}$ with $\lambda_{pre}$ being the free stretch in the thickness direction after the application of an electric field. The electric field in the sample is considered to reach a steady and uniform state quickly once the electric potential difference between the two largest surfaces is applied. Thus, the electric field can be calculated as $E=\frac{∆\phi }{l}$, where ∆ϕ and l are respectively the electric potential difference and the thickness of the VHB4905 sample. Because the relaxation times of the 1st, 2nd, and 3rd subnetworks are, respectively, 413.32 s, 5.43 s, and 1.65 s, we assume that the 1st subnetwork is completely elastic and the 2nd and 3rd subnetworks are completely viscous prior to the mechanical test. Therefore, using $P_{1}=0$ in the case of free expansion, Equation (65) can be reduced to the following:

(68) $$\frac{\left(G^{eq}+G_{1}^{neq}\right)L\left(1/\lambda_{pre}-\lambda_{pre}^{2}\right)}{L-2/\lambda_{pre}-\lambda_{pre}^{2}+3}=0$$

which can be solved to obtain $\lambda_{pre}$. When the sample starts to be stretched, the initial conditions are given by the following:

(69) $$\lambda_{1}=\lambda_{2}=\lambda_{21}^{v}=\lambda_{22}^{v}=\lambda_{31}^{v}=\lambda_{32}^{v}=\frac{1}{\sqrt{\lambda_{pre}}},\;\;\;\;\lambda_{11}^{v}=\lambda_{12}^{v}=1$$

A finite difference approach is employed to obtain the numerical results of the evolving stress $P_{1}$ with the stretch $\lambda_{1}$ by solving the coupled Equations (65) and (67) under the initial conditions (69).

**Figure 9:**
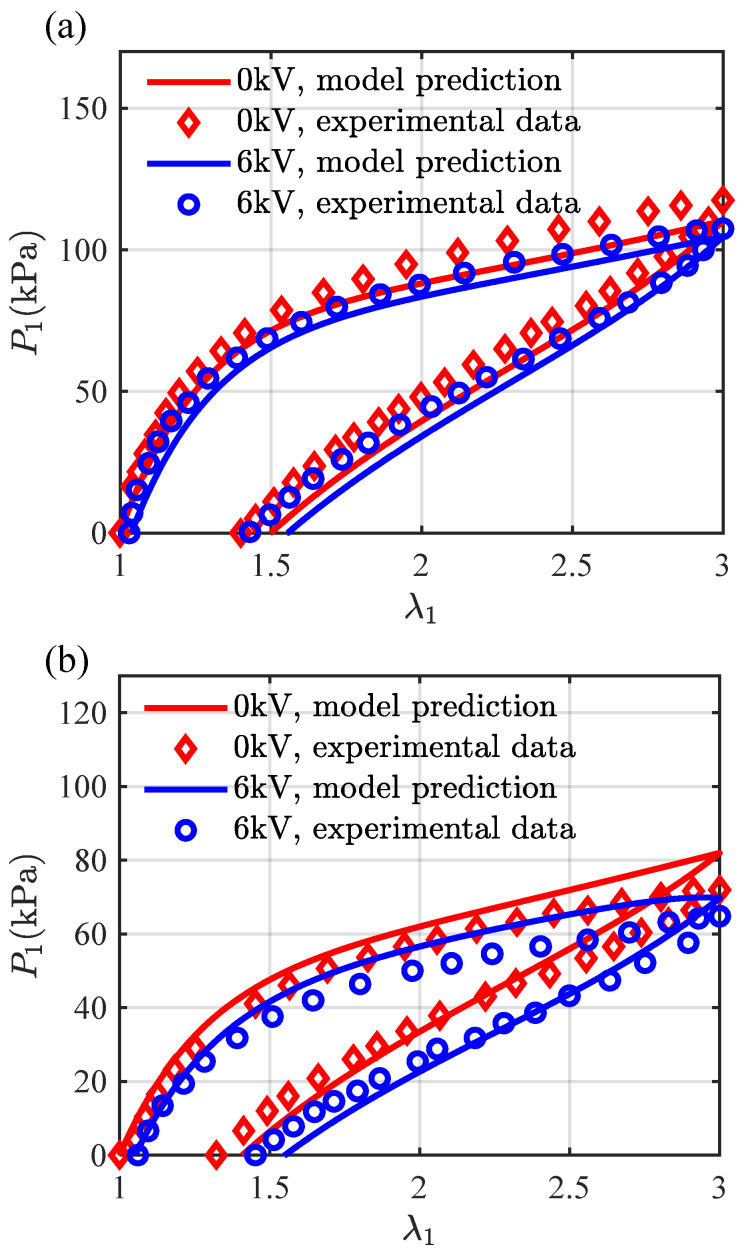
A comparison of the loading-unloading curves from the model prediction and the experimental data at (**a**) 296 K and (**b**) 353 K. The loading-unloading rate is $\left|{\dot{\lambda }}_{1}\right|=0.2/s$, and the applied electric potential difference is 0 kV or 6 kV. The experimental data are taken from Mehnert et al. [21].

Figure 9 gives a comparison of the evolving stress $P_{1}$ with the stretch $\lambda_{1}$ at $\left|{\dot{\lambda }}_{1}\right|=0.2/s$ from the model prediction and the experimental data at different temperatures and electric fields. The equilibrium and non-equilibrium moduli for ϑ=296 K are listed in Table 2 and for ϑ=333 K are calculated via the relations (61) and (62) as $G^{eq}=12.36\;kPa$, $G_{1}^{neq}=12.24\;kPa$, $G_{2}^{neq}=17.38\;kPa$, and $G_{3}^{neq}=20.43\;kPa$. Here, we assume that the material parameters would not change with the electric field in consideration of the relatively small effect of the electric field on the viscous response in the experiments. It can be seen from Figure 9a that the loading-unloading curve with an applied electric potential difference of 6 kV (or an electric field $12\times 10^{6}\;V/m$) does not start exactly at the stretch of 1 but slightly above 1 (the pre-stretch of $\lambda_{1}$ is 1.0383), which captures well the electric field-induced deformation prior to the mechanical deformation in the experiment. The curve without the electric potential is slightly below the experimental data because we have neglected the slight influence of the compliant electrodes on the viscoelastic response investigated by Mehnert and Steinmann [34]. Similarly, in Figure 9b, the pre-stretch of $\lambda_{1}$ for 323 K is 1.0540, and the distinction of the loading-unloading curves with and without an applied electric potential difference is relatively significant. Furthermore, it can be observed from Figure 9a,b that temperature fluctuation has a significantly more pronounced effect on the viscoelastic response than electric field, and the influence of electric field at high temperatures becomes clearly visible. The predicted loading-unloading curves match well with the experimental data, indicating that the present model is able to describe the thermo-electro-viscoelastic response of VHB4905 and has great potential in engineering applications of DEs. 

## 4. Conclusions

In this paper, a thermodynamically consistent model at finite deformation for the thermo-electro-viscoelastic coupling behaviors of DEs is proposed and verified by the comparison of the nonlinear loading-unloading curves from the model prediction and the experimental data of VHB4905. The major novelty of the present work lies in the following aspects: First, we have proposed a thermo-electro-viscoelastic model for DEs at finite deformation, which can describe the nonlinear response of DEs at various stretching rates, temperatures, and electric fields. Especially, the elastic deformation incompressibility of both the ground network and a few parallel subnetworks is considered by introducing Lagrange multipliers, for which the distinct effects of the incompressibility on the force equilibrium and the viscous flow are clearly presented. The incompressibility conditions directly change the magnitude of the stress but have no influence on the viscous flow because only the deviatoric stress, excluding the hydrostatic pressure, promotes the viscous flow. Second, the WLF-like dependence of the moduli on temperature is found by fitting the model with the experimental data, contributing to the calculation of the moduli at different temperatures for theoretical modeling. Third, we also simulate the instant pre-stretch due to the interaction of quasi-static charges after the application of an electric field, which has not been modeled in the previous work.

The numerical results reveal that increasing temperature can soften DEs significantly, and the influence of the electric field on the mechanical response of DEs with respect to temperature is slight. Thus, this work provides a guide on modeling the thermo-electro-mechanical coupling behaviors of DEs and can help analyze the failure of DEs in functionalities and structures. Simultaneously, this theoretical model can be applied to different materials, such as piezoelectric composites, which will be our future study subject.

## Figures and Tables

**Table 1:** Material parameters.

Parameters	Values
$c_{0}\left(\left(J/\left(m^{3}\cdot K\right)\right)\right)$	$8.86\times 10^{5}$
$c_{v}\left(J/\left(m^{3}\cdot K\right)\right)$	$9.18\times 10^{5}$
$\kappa_{0}\left(W/\left(m\cdot K\right)\right)$	0.42
$\kappa_{v}\left(W/\left(m\cdot K\right)\right)$	−0.17
$h\left(W/\left(m^{2}\cdot K\right)\right)$	20

**Table 2:** Material parameters at different temperatures.

	273 K	296 K	313 K	333 K	353 K
$G^{eq}\left(kPa\right)$	23.44	15.12	13.05	11.82	11.07
$G_{1}^{neq}\left(kPa\right)$	38.44	16.83	13.72	11.55	10.20
$G_{2}^{neq}\left(kPa\right)$	94.23	27.39	23.90	13.11	13.29
$G_{3}^{neq}\left(kPa\right)$	94.96	41.04	20.57	25.41	19.49
$\tau_{1}\left(s\right)$	413.32	413.32	413.32	413.32	413.32
$\tau_{2}\left(s\right)$	5.43	5.43	5.43	5.43	5.43
$\tau_{3}\left(s\right)$	1.65	1.65	1.65	1.65	1.65
$\;L,L_{1},L_{2},L_{3}$	155	155	155	155	155

## Data Availability

The data that support the findings of this study are available from the corresponding author upon reasonable request.

## References

[1] Romasanta L.J., Lopez-Manchado M.A., Verdejo R. (2015). Increasing the performance of dielectric elastomer actuators: A review from the materials perspective. Prog. Polym. Sci..

[2] Shi Y., Askounis E., Plamthottam R., Libby T., Peng Z., Youssef K., Pu J., Pelrine R., Pei Q. (2022). A processable, high-performance dielectric elastomer and multilayering process. Science.

[3] Li T., Li G., Liang Y., Cheng T., Dai J., Yang X., Liu B., Zeng Z., Huang Z., Luo Y. (2017). Fast-moving soft electronic fish. Sci. Adv..

[4] Li G., Chen X., Zhou F., Liang Y., Xiao Y., Cao X., Zhang Z., Zhang M., Wu B., Yin S. (2021). Self-powered soft robot in the Mariana Trench. Nature.

[5] Qiu Y., Zhang E., Plamthottam R., Pei Q. (2019). Dielectric elastomer artificial muscle: Materials innovations and device explorations. Acc. Chem. Res..

[6] Chuc N.H., Vuong N.H.L., Kim D., Moon H., Koo J.C., Lee Y., Nam J.-d., Choi H.R. (2012). Design and control of a multi-jointed robot finger driven by an artificial muscle actuator. Adv. Robot..

[7] Lin G.J., Zhang X.B., Song D.C. (2011). Wind power micro-generator using dielectric electric active polymer. Adv. Mater. Res..

[8] Moretti G., Papini G.P.R., Righi M., Forehand D., Ingram D., Vertechy R., Fontana M. (2018). Resonant wave energy harvester based on dielectric elastomer generator. Smart Mater. Sruct..

[9] Rosset S., Shea H.R. (2012). Flexible and stretchable electrodes for dielectric elastomer actuators. Appl. Phys. A.

[10] Marette A., Poulin A., Besse N., Rosset S., Briand D., Shea H. (2017). Flexible zinc-tin oxide thin film transistors operating at 1 kV for integrated switching of dielectric elastomer actuators arrays. Adv. Mater..

[11] Suo Z. (2010). Theory of dielectric elastomers. Acta Mech. Solida Sin..

[12] Khurana A., Joglekar M.M., Zurlo G. (2022). Electromechanical stability of wrinkled dielectric elastomers. Int. J. Solids Struct..

[13] Ghosh A., Basu S. (2021). Soft dielectric elastomer tubes in an electric field. J. Mech. Phys. Solids.

[14] Guo Y., Liu L., Liu Y., Leng J. (2021). Thermoelectromechanical instability of dielectric elastomer undergoes polarization saturation and temperature variation. Acta Mech. Sin..

[15] Sheng J., Chen H., Li B. (2011). Effect of temperature on the stability of dielectric elastomers. J. Phys. D Appl. Phys..

[16] Plante J.-S., Dubowsky S. (2006). Large-scale failure modes of dielectric elastomer actuators. Int. J. Solids Struct..

[17] Diaconu I., Dorohoi D.-O., Ciobanu C. (2008). Electromechanical response of polyurethane films with different thickness. Rom. J. Phys..

[18] Hossain M., Vu D.K., Steinmann P. (2014). A comprehensive characterization of the electro-mechanically coupled properties of VHB 4910 polymer. Arch. Appl. Mech..

[19] Hossain M., Vu D.K., Steinmann P. (2012). Experimental study and numerical modelling of VHB 4910 polymer. Comp. Mater. Sci..

[20] Liao Z., Hossain M., Yao X., Mehnert M., Steinmann P. (2020). On thermo-viscoelastic experimental characterization and numerical modelling of VHB polymer. Int. J. Non-Linear Mech..

[21] Mehnert M., Hossain M., Steinmann P. (2021). A complete thermo–electro–viscoelastic characterization of dielectric elastomers, Part I: Experimental investigations. J. Mech. Phys. Solids.

[22] Hong W. (2011). Modeling viscoelastic dielectrics. J. Mech. Phys. Solids.

[23] Behera S.K., Kumar D., Sarangi S. (2021). Modeling of electro–viscoelastic dielectric elastomer: A continuum mechanics approach. Eur. J. Mech. A/Solids.

[24] Chen Y., Kang G., Yuan J., Hu Y., Li T., Qu S. (2020). An electro-mechanically coupled visco-hyperelastic-plastic constitutive model for cyclic deformation of dielectric elastomers. Mech. Mater..

[25] Zhou J., Jiang L., Khayat R.E. (2018). A micro–macro constitutive model for finite-deformation viscoelasticity of elastomers with nonlinear viscosity. J. Mech. Phys. Solids.

[26] Mehnert M., Hossain M., Steinmann P. (2019). Experimental and numerical investigations of the electro-viscoelastic behavior of VHB 4905TM. Eur. J. Mech. A. Solids.

[27] Alkhoury K., Bosnjak N., Wang Y., Lee H., Nadimpalli S., Chester S.A. (2022). Experiments and modeling of the thermo-mechanically coupled behavior of VHB. Int. J. Solids Struct..

[28] Mehnert M., Hossain M., Steinmann P. (2021). A complete thermo-electro-viscoelastic characterization of dielectric elastomers, Part II: Continuum modeling approach. J. Mech. Phys. Solids.

[29] Gent A.N. (1996). A new constitutive relation for rubber. Rubbery Chem. Technol..

[30] Banks H.T., Hu S., Kenz Z.R. (2015). A brief review of elasticity and viscoelasticity for solids. Adv. Appl. Math. Mech..

[31] Reese S., Govindjee S. (1998). A theory of finite viscoelasticity and numerical aspects. Int. J. Solids Struct..

[32] Su X., Peng X. (2018). A 3D finite strain viscoelastic constitutive model for thermally induced shape memory polymers based on energy decomposition. Int. J. Plast..

[33] Qin B., Zhong Z., Zhang T.-Y. (2022). A thermodynamically consistent model for chemically induced viscoelasticity in covalent adaptive network polymers. Int. J. Solids Struct..

[34] Mehnert M., Steinmann P. (2019). On the influence of the compliant electrodes on the mechanical behavior of VHB 4905. Comp. Mater. Sci..

[35] Arruda E.M., Boyci M.C. (1993). A three-dimensional constitutive model for the large stretch behaviour of rubber elastic materials. J. Mech. Phys. Solids.

[36] Ganser M., Hildebrand F.E., Kamlah M., McMeeking R.M. (2019). A finite strain electro-chemo-mechanical theory for ion transport with application to binary solid electrolytes. J. Mech. Phys. Solids.

[37] N’Guyen T.A., Lejeunes S., Eyheramendy D., Boukamel A. (2016). A thermodynamical framework for the thermo-chemo-mechanical couplings in soft materials at finite strain. Mech. Mater..

[38] Dippel B., Johlitz M., Lion A. (2015). Thermo-mechanical couplings in elastomers—Experiments and modelling. ZAMM J. Appl. Math. Mech..

[39] Kollosche M., Kofod G., Suo Z., Zhu J. (2015). Temporal evolution and instability in a viscoelastic dielectric elastomer. J. Mech. Phys. Solids.

[40] Shangguan Y., Chen F., Jia E., Lin Y., Hu J., Zheng Q. (2017). New insight into time-temperature correlation for polymer relaxations ranging from secondary relaxation to terminal flow: Application of a universal and developed WLF equation. Polymers.

